# Whole Body Protein Oxidation Unaffected after a Protein Restricted Diet in Healthy Young Males

**DOI:** 10.3390/nu11010115

**Published:** 2019-01-08

**Authors:** Gerlof A.R. Reckman, Gerjan J. Navis, Wim P. Krijnen, Cees P. van der Schans, Roel J. Vonk, Harriët Jager-Wittenaar

**Affiliations:** 1 Department of Internal Medicine, Division of Nephrology, University of Groningen, University Medical Center Groningen, AA53, PO Box 30.001, 9700 RB Groningen, The Netherlands; g.a.r.reckman@pl.hanze.nl; 2 Research Group Healthy Ageing, Allied Health Care and Nursing, Centre of Expertise Healthy Ageing, Hanze University of Applied Sciences, Petrus Driessenstraat 3, 9714 CA Groningen, The Netherlands; w.p.krijnen@pl.hanze.nl (W.P.K.); c.p.van.der.schans@pl.hanze.nl (C.P.v.d.S.); 3 Department of Internal Medicine, Division of Nephrology, University of Groningen, University Medical Center Groningen, AA53, PO Box 30.001, 9700 RB Groningen, The Netherlands; g.j.navis@umcg.nl; 4 Department of Rehabilitation and Health Psychology, University of Groningen, University Medical Center Groningen, CD44, PO Box 30.001, 9700 RB Groningen, The Netherlands; 5 Department of Cell Biology, University of Groningen, University Medical Center Groningen, FB33, PO Box 30.001, 9700 RB Groningen, The Netherlands; r.j.vonk@umcg.nl; 6 Department of Maxillofacial Surgery, University of Groningen, University Medical Center Groningen, BB70, PO Box 30.001, 9700 RB Groningen, The Netherlands

**Keywords:** Protein, oxidation, anabolic competence, breath test, naturally enriched ^13^C-milk proteins

## Abstract

Protein oxidation may play a role in the balance between anabolism and catabolism. We assessed the effect of a protein restricted diet on protein oxidation as a possible reflection of whole body protein metabolism. Sixteen healthy males (23 ± 3 years) were instructed to use a 4-day isocaloric protein restricted diet (0.25 g protein/kg body weight/day). Their habitual dietary intake was assessed by a 4-day food diary. After an overnight fast, a 30 g ^13^C-milk protein test drink was administered, followed by 330 min breath sample collection. Protein oxidation was measured by Isotope Ratio Mass Spectrometry. To assess actual change in protein intake from 24-h urea excretion, 24-h urine was collected. During the 4-day protein restricted diet, the urinary urea:creatinine ratio decreased by 56 ± 9%, which is comparable to a protein intake of ~0.65 g protein/kg body weight/day. After the protein restricted diet, 30.5 ± 7.3% of the 30 g ^13^C-milk protein was oxidized over 330 min, compared to 31.5 ± 6.4% (NS) after the subject’s habitual diet (1.3 ± 0.3 g protein/kg body weight/day). A large range in the effect of the diet on protein oxidation (−43.2% vs. +44.0%) was observed. The residual standard deviation of the measurements was very small (0.601 ± 0.167). This suggests that in healthy males, protein oxidation is unaffected after a protein restricted diet. It is uncertain how important the role of fluctuations in short-term protein oxidation is within whole body protein metabolism.

## 1. Introduction

Adequate protein intake and subsequent utilization of protein is of great importance for health. The recommended daily protein intake for healthy adults is 0.8 g protein/kg body weight/day, and is suited for maintaining normal body composition and meeting metabolic demand [1]. Patients with disease-related malnutrition (DRM) have an absolute or relative deficiency and inadequate utilization of energy, protein, and other nutrients caused by a concomitant disease. Compromised outcomes, such as impaired clinical outcome from disease, and diminished physical and mental function have been described in relation to DRM [2,3,4]. Nutrition, exercise, and the hormonal milieu are essential to reach a state which optimally supports protein synthesis and lean body mass (LBM), global aspects of muscle and organ function, and the immune response, a paradigm also known as “anabolic competence” [5].

Prevention and treatment of LBM loss could benefit from direct measurement and monitoring of disturbed protein metabolism. Current methods to measure protein metabolism focus on protein synthesis, which requires blood sampling, muscle biopsies, and/or the use of expensive synthetic labelled amino acids [6,7,8]. Therefore, these methods are not suitable for the clinical setting. A non-invasive bedside method to measure protein metabolism would be more suitable, as direct measurements of the metabolic state could lead to more insight in optimal protein intake and optimal physical activity, which then enables tailored improved treatment for each patient, resulting in improved outcomes of disease.

Measuring protein oxidation is a feasible and non-invasive technique and can be performed with naturally labelled ^13^C-protein, which is relatively inexpensive. All oxidized ^13^C-protein will be exhaled as ^13^CO_2_ [9]. However, it is unknown to what extent variations in protein oxidation occur under various physiological conditions, such as changes in protein intake. Generally, after the ingestion of protein, the protein derived amino acids will be incorporated into new proteins until protein synthesis requirements are met. The lack of protein storage leads to the oxidation of surplus amino acids [10,11]. Accordingly, an altered protein intake could modify protein oxidation under normal conditions. Thus, we hypothesized that restriction in protein intake in healthy subjects leads to decreased activity of the oxidation pathway, as assessed by the ^13^CO_2_ breath test.

To test this hypothesis, in the current study, we aimed to measure the effect of a four-day protein restricted diet, compared to their habitual diet, on protein oxidation, as assessed by the ^13^CO_2_ breath test in healthy subjects.

## 2. Materials and Methods

### 2.1. Subjects

Healthy young males were included as being a representative group for healthy subjects. The decision to recruit young subjects versus older subjects was based on logistics, as the pool of young healthy subjects is more easily accessible for study. Women were excluded to rule out possible effects of the menstrual cycle on protein metabolism, and to exclude possible effects of differences in body composition between women and men. Furthermore, subjects having a disease and/or undergoing or starting medical treatment were excluded. Sixteen healthy young male subjects were recruited via local advertising. To obtain a homogeneous group of subjects, reducing the possible influence of covariates, the following inclusion criteria were applied: Age between 18–30 years, body mass index (BMI) between 20–25 kg/m^2^, and being able to fast overnight. Exclusion criteria were: Having a disease and/or being medically treated, milk protein allergy or intolerance, smoking, use of drugs, drinking on average more than 2 glasses of alcohol per day, waist circumference larger than 102 cm, and using a vegetarian diet. This design was chosen to minimize possible confounding effects of subject characteristics over the protein restricted diet intervention.

The study was approved by the local Medical Ethical Committee at the University Medical Center Groningen (NL56982.042.16, METc 2016.144), conducted in accordance with the Helsinki Declaration of 2013, and registered in the Dutch Trial Register under the registration number, NTR6101. After receiving an information letter about the purpose and practical procedures of the study, and an informative meeting with the researcher, every subject gave his written informed consent prior to participation.

### 2.2. Study Protocol

In each subject, age (year), height (cm), waist circumference (cm), bodyweight (kg), BMI (kg/m^2^), and LBM (kg) were measured. LBM was measured by bioelectrical impedance analysis (Quadscan 4000, Bodystat Ltd., Isle of Man, British Isles). After these measurements, subjects were instructed to keep a four-day food diary with respect to their habitual food intake to calculate the average daily intake of energy (kcal), protein (g), protein, en%, animal protein (g), plant protein (g), carbohydrates (g), carbohydrates, en%, fat (g), and fat, en%. The calculations on dietary intake were performed with Evry (Evry BV), which uses the NEVO 2013, RIVM database [12].

On each subject, at two separate days, two breath tests were performed; one after the subject’s habitual diet and one after an isocaloric protein restricted diet (0.25 g protein/kg body weight/day). Between the breath tests, there was a washout period of at least a week to return to baseline ^13^CO_2_ levels. On the evening before the breath test, subjects were instructed to start fasting overnight (only consumption of water and tea, or coffee without milk and sugar was allowed) from 22:00 p.m. onwards to arrive sober the next morning at 08:45 a.m. During each test, 3 basal breath samples were collected and averaged to establish the subject’s baseline ^13^CO_2_:^12^CO_2_ ratio. At 09:15 a.m., 30 g naturally enriched ^13^C-milk protein dissolved in 500 mL water was consumed within 5 minutes. The isotope, ^13^C, is a stable isotope. From 09:25 a.m. until 14:45 p.m. (5.5 h), a breath sample was collected every 10 minutes. During this period, subjects were instructed to remain seated in upright position and not to eat or drink during the remainder of the breath test. Subjects were allowed to work on a laptop, to read, and to write.

The four-day protein restricted diet was given as a food menu, which described in detail what and when to eat, to facilitate energy and protein intake as prescribed. The subjects were instructed to use the food menus. The food menu was tailored to each subject’s habitual energy intake, which was calculated from the four-day food diaries. Therefore, the created protein restricted diet was isocaloric to the habitual diet. Consequently, by both reducing protein intake and keeping the protein restricted diet isocaloric, the macronutrient composition of the diet changed, as the energy lost from protein intake was replaced by mainly an increase in carbohydrates and to a lesser extent with an increase in fat, as food which contains fat also contains protein. Each subject underwent the breath tests in the same order: Starting the first after habitual diet and second after the protein restricted diet. The four-day protein restricted diet was tailored to each subject.

During five days, 24-h urine was collected, on the fourth day of the subject’s habitual diet and, next, every day during the four-day isocaloric protein restricted diet. From each 24-h urine sample, urea and creatinine concentrations were measured to calculate the subject’s actual protein intake [13]. The urea:creatinine ratio was calculated to assess the compliance to the isocaloric protein restricted diet. A change to a protein restricted diet will reduce 24-h urinary urea production, while 24-h urinary creatinine production, which depends almost exclusively on muscle mass, will remain steady, and therefore the urea:creatinine ratio will decrease.

### 2.3. Calculations and Statistical Analysis

Disturbances in short-term protein oxidation can be measured by pulse-labelling with the use of naturally enriched ^13^C-proteins. After ingestion of ^13^C-proteins, the protein oxidation can be quantified by measuring the ^13^CO_2_:^12^CO_2_ ratio in exhaled breath over time. To calculate the amount of CO_2_ produced on each timepoint, the CO_2_ production in rest was calculated by the following regression formula: 300 mmol CO_2_/hour × body surface area (BSA), which was calculated with the Haycock formula [14]:

(1) BSA (m^2)= weight (kg)^0.5378 ×height (cm)^0.3964 × 0.024265

The breath samples were measured for their ^13^CO_2_:^12^CO_2_ ratio with an isotope ratio mass spectrometer (IRMS) and compared to a high ^13^C-enriched international standard, Pee Dee Belemnite (PDB), which has an accepted absolute ^13^C/^12^C ratio of 0.0112372. The differences (delta, δ) between the breath samples and the standard is expressed in parts per 1000 (‰) as [15] follows:

(2) δ 13C sample=((13C/12Csample)/(13C/12Cstandard)−1)×1000

The PDB standard ^13^C/^12^C ratio is defined as 0‰. To calculate the ^13^C/^12^C ratio from the IRMS delta values, the following inversion formula was used [10]:

(3) 13C/12Cratio=((deltavalue/1000)+1)×0.0112372

Next, the ^13^C/^12^C ratio of each breath sample is used to calculate the %^13^C [16] by:

(4) %13C=((13C/12Cratio)/(13C/12Cratio+1))×100

The baseline (*t* = 0) breath sample ^13^C/^12^C ratio was subtracted from each subsequent breath sample to acquire the change from the subject’s baseline. The estimated CO_2_ production, together with the delta value on each timepoint, and the enrichment of the ^13^C-milk protein was used to calculate the protein oxidation rate of the subject at each timepoint with 10 minutes in between.

The change in the ^13^CO_2_:^12^CO_2_ ratio over time has been described [17,18] by the general concentration model as follows:

(5) y(t)=a× t^b×e^(−kt) + ε (normally distributed)

The model function was fitted to the measurement data for each subject over time, with *t* ranging from zero to 330 minutes. That is, the parameters, *a*, *b*, and *k*, were determined by fitting the oxidation rate curves to the measurement data per subject per day, with the goal of finding optimal parameters per subject per day. Total protein oxidation was calculated as the integral over the curve representing the area under the curve (AUC). The term, *ε*, is normally distributed with a zero mean, and its variance is estimated by the residual error variance. Each resulting curve starts at the natural amount, *y* = 0, as no ^13^C-milk protein has been ingested at timepoint, *t* = 0. After ingestion, the stomach releases the ^13^C-milk protein into the digestive track where the proteins are digested and taken up by the gut. The digested proteins are circulated and become available to the cells for protein synthesis or oxidation. The process of protein oxidation is reflected by the oxidation curve. From *t* = 0 onwards, the oxidation curve ascends, reaching its maximum and thereafter, the oxidation rate of ^13^C-amino acids descends towards the subject’s baseline over multiple hours.

Per breath test, the values of the fitted parameters, *a*, *b*, and *k*, differ over persons and type of diet. For larger values of *a* and *b*, the ascending slope becomes steeper, whereas a higher value for the constant, *k*, leads to *a* steeper descending slope after the maximum was reached.

The difference in the average AUC after a habitual diet and protein restricted diet within subjects was tested with the paired *t*-test. Two other important characteristics directly calculated from the parameters of each fitted curve per subject were the timepoint (*t*_max_) in minutes at which the maximum oxidation rate was reached, as well as the corresponding maximum oxidation rate, *y*(*t*_max_), where *t*_max_ = *b*/*k*. The latter was expected to be similar within subjects in both experimental conditions, as *t*_max_ is mainly determined by the rate of stomach emptying [19], which is dependent on the test drink. The latter was identical for both breath tests. The standard error of the *t*_max_ per person is computed by the delta method [20]. Both the maximum oxidation rate, *y*(*t*_max_), and the total oxidation (AUC) were expected to be lower after the protein restricted diet compared to the habitual diet, as a deficit of amino acids in the body is hypothesized to lead to less oxidation of the 30 g ingested milk protein. From each concentration model, the timepoint at which 1% oxidation/hour was reached was calculated using the above formula and a numerical intersection method.

All statistical analyses were performed by the statistical programming language, R (R Core Team, 2017), with the package, “car” [21], specifically using the non-linear least squares function [22] to fit the concentration curve to the measurements over time and the delta method to determine the standard error of *t*_max_ [20]. The difference in the urea:creatinine ratio between the habitual diet and day four of the protein restricted diet was investigated with the paired Student’s *t*-test. The associations between total protein oxidation and the demographic characteristics, i.e., age, bodyweight, BMI, LBM, habitual protein intake, habitual energy intake, and baseline urea:creatinine ratio, were investigated with the Pearson correlation coefficient, *r*. All data are represented as mean ± standard deviation (SD).

## 3. Results

Baseline characteristics of the 16 male subjects are presented in Table 1.

**Figure 1:**
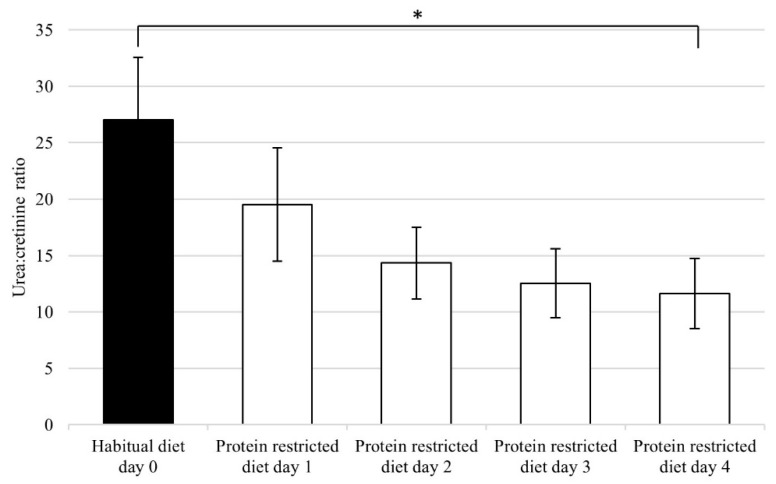
Urea:creatinine ratio calculated from 24-h urine collections (*n* = 16). The symbol “*” denotes the statistically significant change from day 0 to day 4 with *p* < 0.001. Protein intake during the habitual diet was 1.3 g protein/kg body weight/day ± 0.3 g; the prescribed four-day protein restricted diet was 0.25 g protein/kg body weight/day

During the four-day protein restricted diet, the urea:creatinine ratio in 24-h urine decreased with an average of 56 ± 9%, as compared to the habitual diet from day 0 to day 4 (Figure 1). The mean difference in the urea:creatinine ratio between the habitual diet day 0 and protein restricted diet day 4 was statistically significant (*p* < 0.001, *t* = 12.837, *df* = 15). Based on the change in the urea:creatinine ratio, the protein intake decreased to 0.65 g protein/kg body weight/day, compared to the habitual protein intake of 1.3 g protein/kg body weight/day.

**Figure 2:**
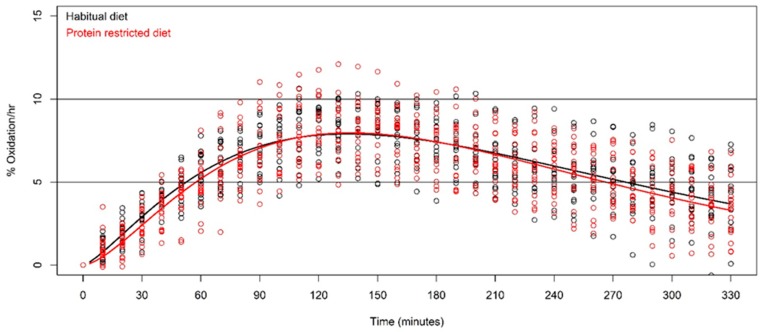
Protein oxidation kinetics after the habitual diet (black) and protein restricted diet (red) (*n* = 16).

The average protein oxidation kinetics of all subjects during the 330 minute breath tests, separated by the habitual diet and the protein restricted diet, are shown in Figure 2. Total oxidation (AUC) after the habitual diet and the protein restricted diet was 31.5 ± 6.4% and 30.5 ± 7.3%, respectively. The difference in the mean total oxidation between the habitual and protein restricted diet was not statistically significant (*p* = 0.530, *t* = 0.643, *df* = 15). The mean total protein oxidation of ~30%, corresponds to ~10 g oxidized. Time to *t*_max_, after the habitual and protein restricted diet, was 137 minutes ± 24 and 138 minutes ± 18, respectively (*p* = 0.854, *t* = −0.188, *df* = 15). The maximum %oxidation rate per hour, after the habitual diet and the protein restricted diet, was 8.05 ± 1.27% and 8.11 ± 1.62% (*p* = 0.868, *t* = −0.170, *df* = 15).

The following means and confidence intervals were calculated with all 32 breath tests (16 subjects × 2 breath tests). From the curve fitting with the function, *y(t) = a × t^b × e^(−kt)*, the mean of constant, *a*, was 0.037 ± 0.041. For the constant, *b*, the mean was 1.636 ± 0.458. For the constant, *k*, the mean was 0.012 ± 0.004. The concentration curve fitted well with the breath test measurements with a mean *R*^2^ of 0.930 ± 0.033. The timepoint of maximum oxidation (*t*_max_) was obtained with a mean 137 ± 21 minutes. The mean proportionate decrease from the maximum oxidation rate (mean 8.1% oxidation/hour) to the final timepoint (mean 3.5% oxidation/hour) over all 32 breath tests was −57 ± 16%. The mean time to reach 1% oxidation/hour was 502 ± 119 minutes. The total oxidation was positively correlated with the maximal oxidation rate (0.95). Residual standard deviation of the measurements to the fitted curve was 0.601 ± 0.167. The associations between the total protein oxidation and demographic characteristics, i.e., age, body weight, BMI, LBM, habitual protein intake, habitual energy intake, and baseline urea:creatinine ratio, were fair to poor (*r* < 0.4) [23].

**Figure 3:**
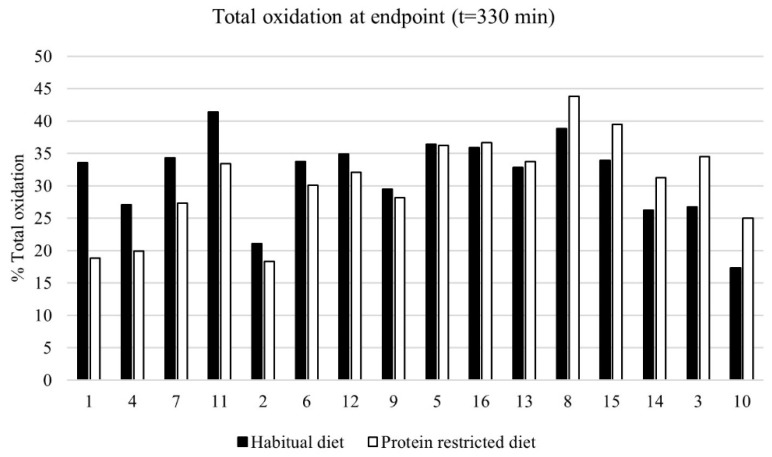
Total protein oxidation (% of given 30 g dose) measured with the breath test after a habitual diet (black bars) versus the protein restricted diet (0.25 g/kg body weight/day)(white bars) (*n* = 16). Subjects are ordered from left to right, based on the strongest relative reduction in protein oxidation from their habitual diet to the protein restricted diet, towards the strongest relative increase.

Differences in the total protein oxidation after each subject’s habitual diet (1.3 ± 0.3 g protein/kg body weight/day), and after a prescribed isocaloric protein restricted diet (0.25 g/kg body weight/day) are shown in Figure 3. The results of the subjects are ordered based on the strongest relative decrease in protein oxidation from their habitual diet to the protein restricted diet, towards the strongest relative increase. A large range in the effect on the total protein oxidation (relative change −43.2% vs. +44.0%) was observed, however, the large range of the increase and decrease in the total protein oxidation canceled each other out, as shown in Figure 2.

## 4. Discussion

In the current study, we assessed the effect of a protein restricted diet on protein oxidation, as assessed by the ^13^CO_2_ breath test in healthy young males, as a possible reflection of whole body protein metabolism. On the group level, the total protein oxidation was not affected by the two-fold reduction in mean protein intake, which decreased from 1.3 to 0.65 g protein/kg body weight/day. We found a large variation in the total protein oxidation response after the protein restricted diet compared to the habitual diet, which ranged from a decrease of 43.2% to an increase of 44.0%. Combined with the precision of the breath test, this implies that the range in effects on the oxidation rate may have a biological background.

This is the second study that has assessed the overall protein oxidation with naturally ^13^C-enriched milk protein during different states of protein intake. An explorative investigation was performed with a three-day protein restricted isocaloric diet, in which the protein intake was reduced to ~10 g of protein per day, which corresponds to ~0.15 g protein/kg body weight/day [9]. In that study, the total protein oxidation after the protein restricted diet had a lower mean than after the habitual diet, but the difference in the mean was not significant (*p* = 0.142). Further comparison of our results with those of other studies, which either measured the effect of several conditions or the effect of different exercise regimens combined with protein ingestion on the protein synthetic response, is difficult. However, our finding that ingestion of 30 g of ^13^C-milk protein was associated with an overall oxidation of ~30%, corresponding to ~10 g oxidation over 5.5 hours, seems in line with the study by Moore et al. [8]. In that study, in which protein muscle synthesis was measured with primed ^13^C-leucine infusion over 4 hours, muscle protein synthesis after resistance exercise was maximally stimulated with 20 g of ingested whole egg protein and dietary protein ingested in excess of 20 g stimulated ^13^C-leucine oxidation. Therefore, Moore et al. imply that they would have found ~10 g of whole egg protein oxidized if they had tested a 30 g dose of whole egg protein, which is comparable to the amount of ^13^C-milk protein oxidized in our study. Our findings did not confirm our hypothesis that a change in protein intake would be associated with a change in overall protein oxidation. This could mean that a change in protein intake in the range investigated here does indeed not affect protein oxidation in healthy males, or, alternatively, that there were methodological limitations in detecting such an alleged change in the current methodological design of our study.

The first limitation that needs to be taken into account is that there could be a pre-meal effect of the non-standardized evening meal, prior to the breath test, on the utilization and oxidation subsequent to the 30 g protein test drink. However, no relationship between the evening meal energy intake, en% carbohydrates, and en% protein intake on the subsequent protein oxidation was found, which could in part be related to the small sample size. Second, during the protein restricted diet, the subjects did not reach the intended target of 0.25 g protein/kg body weight/day intake, as the mean level of protein intake decreased to only 0.65 g protein/kg body weight/day as measured by the urea:creatinine ratio. This level of intake is only slightly lower than the general protein intake recommendation of 0.80 g/kg body weight/day for healthy adults set by the World Health Organization (WHO) [1]. It would seem that in this study, the subjects were probably still within the range of adequate protein intake to maintain protein homeostasis. On the other hand, whether the subjects in this study were in steady state is uncertain, as they acutely adjusted their protein intake from habitual to the four-day protein restricted diet. The use of 24-hour urinary urea for the estimation of dietary protein intake is most reliable in subjects who are in a steady state [24,25]. Therefore, it could be argued that in these experimental circumstances, the change in the urea:creatinine ratio does not accurately reflect protein homeostasis and also the actual protein intake. However, it does at least underline a clear reduction in protein intake. Third, the replacement of protein with carbohydrates to obtain an isocaloric protein restricted diet changed the macronutrient ratios within the diet, and might have resulted in a decreased uptake of amino acids into tissue and, consequently, an increased amino acid oxidation in the splanchnic area due to a possible altered insulin response [26,27]. Fourth, the current protocol includes an overnight fast, to forgo breakfast, and after consumption of the test drink, the subjects did not eat or drink for 5.5 hours. This was in order to minimize the influence of differences in starting conditions, such as the stomach emptying rate, between the subjects. These requirements enable better interpretable measurements and are unlikely to harm healthy subjects. However, fasting can further deteriorate the condition of clinical populations. For future studies in clinical populations, adaptations to the protocol should be made to minimize the burden. Potential targets to reduce the burden are a standardized breakfast and reduction in the collection of breath samples over time.

The major strengths of this study were the well-controlled design, with subjects as their own control, and the precise protein oxidation measurements. First, in the current tests, natural enriched ^13^C-milk protein was used, which implies that all amino acids are enriched with ^13^C and therefore the exhaled ^13^CO_2_ represents the oxidation of all amino acids, which reflects the total body protein oxidation [10]. Oxidation studies with specific amino acids, like ^13^C-leucine, most likely do not reflect overall amino acid oxidation, as all the amino acids have various biological functions, aside from being building blocks for synthesizing protein [28]. Second, the breath test is reliable as it measures the protein oxidation process well, with the concentration function fitting well to all breath test measurements, as demonstrated by a mean R^2^ of 0.930 ± 0.033. The formula of each curve provided estimated parameters per subject. These parameters, such as the timepoint of the maximum, and the oxidation rate at the timepoint of the maximum, had small standard errors. Moreover, the residual standard deviation of the oxidation rate was very small (0.601 ± 0.167). As the breath test is reliable, the personal parameters found have a biological basis. Finding potentially important biological factors involved in protein oxidation and protein utilization are a next step in understanding whole body protein metabolism.

In conclusion, this study has shown that on the group level, the total protein oxidation was not affected by a short-term reduction in protein intake in healthy subjects. This suggests that over the range of protein intake investigated here, the overall protein metabolism is robust against challenges. However, due to large variations found on the individual level with respect to the change in total protein oxidation between the habitual and protein restricted diet, and the poor to fair associations of total protein oxidation with demographic characteristics, it is uncertain how important the role of fluctuations in short-term protein oxidation is within whole body protein metabolism.

## Figures and Tables

**Table 1:** Baseline characteristics of the subjects (*n* = 16).

	Mean	SD
Age (years)	23.0	3.1
Height (cm)	185.4	8.6
Body weight (kg)	77.1	9.5
Body Mass Index (kg/m^2^)	22.3	1.1
Lean Body Mass (%)	88.3	2.7
Habitual diet		
Protein intake (g protein/kg body weight/day)	1.3	0.3
Protein intake (g protein/day)	102	25
En% protein (%)	17	4
En% carbohydrates (%)	47	5
En% mono- and disaccharides (%)	20	8
En% fat (%)	35	6
En% saturated fat (%)	13	4
En% unsaturated fat (%)	19	7
Protein restricted diet		
En% protein (%)	3	1
En% carbohydrates (%)	73	7
En% mono- and disaccharides (%)	53	8
En% fat (%)	22	7
En% saturated fat (%)	9	5
En% unsaturated fat (%)	12	6
Baseline breath ^13^CO_2_ enrichment (delta value)	−26.18	0.50
